# Assessment of a ^18^F-Phenylboronic Acid Radiotracer for Imaging Boron in Maize

**DOI:** 10.3390/ijms21030976

**Published:** 2020-02-01

**Authors:** Alexandra B. Housh, Michaela S. Matthes, Amber Gerheart, Stacy L. Wilder, Kun-Eek Kil, Michael Schueller, James M. Guthrie, Paula McSteen, Richard Ferrieri

**Affiliations:** 1 Missouri Research Reactor Center, University of Missouri, Columbia, MO 65211, USA; afbkhn@mail.missouri.edu (A.B.H.); gerheart@msu.edu (A.G.); wildersl@missouri.edu (S.L.W.); schuellerm@missouri.edu (M.S.); guthriejm@missouri.edu (J.M.G.); 2 Chemistry Department, University of Missouri, Columbia, MO 65211, USA; 3 Division of Biological Sciences, Interdisciplinary Plant Group, University of Missouri, Columbia, MO 65211, USA; mathesm@missouri.edu (M.S.M.); mcsteenp@missouri.edu (P.M.); 4 Missouri Maize Center, University of Missouri, Columbia, MO 65211, USA; 5 Department of Veterinary Medicine and Surgery, Columbia, MO 65211, USA; kilk@missouri.edu; 6 Bond Life Sciences Center, University of Missouri, Columbia, MO 65211, USA; 7 Division of Plant Sciences, Interdisciplinary Plant Group, University of Missouri, Columbia, MO 65211, USA

**Keywords:** boron, phenylboronic acid, maize, radiotracer

## Abstract

Boron (B) is an essential plant micronutrient. Deficiencies of B have drastic consequences on plant development leading to crop yield losses and reductions in root and shoot growth. Understanding the molecular and cellular consequences of B deficiency is challenging, partly because of the limited availability of B imaging techniques. In this report we demonstrate the efficacy of using 4-fluorophenylboronic acid (FPBA) as a B imaging agent, which is a derivative of the B deficiency mimic phenylboronic acid (PBA). We show that radioactively labelled [^18^F]FPBA (t_½_=110 m) accumulates at the root tip, the root elongation zone and at lateral root initiation sites in maize roots, and also translocates to the shoot where it accumulates along the leaf edges. Treatment of maize seedlings using FPBA and PBA causes a shortened primary root phenotype with absence of lateral roots in a dose-dependent manner. The primary root defects can be partially rescued by the addition of boric acid indicating that PBA can be used to induce B deficiency in maize and that radioactively labelled FPBA can be used to image sites of B demand on a tissue level.

## 1. Introduction

The trace mineral boron (B) is an essential micronutrient required for plant fitness and crop yield [1,2], as well as animal and human health [3]. B is a weak Lewis acid and is accessible to the plant in form of boric acid (BA) and as the borate anion (BO_3_^3−^). Deficiency of B in the soil is a factor affecting the crop yield worldwide and is reported to be the most widespread micronutrient deficiency [4]. Yet knowledge about the underlying causes and the cellular functions of B is still limited [5]. The best characterized cellular function of B is the crosslinking of pectic subunits, called Rhamnogalacturonan-II (RG-II), in the cell wall [6,7,8]. Cell wall crosslinking by B is needed for proper cell wall stability and plant development [9]. B binding sites in the cell wall are limited and the observation that B levels in the cell wall do not change with altered B levels in the media, while cytosolic levels do, suggests additional roles of B beyond that of the cell wall crosslinking [10]. Indeed, additional roles of B in sugar transport, transpiration, lignification, the metabolism of carbohydrates, RNA, indole-3-acetic acid (IAA), phenols, and as a membrane component have been suggested and demonstrated [11,12].

Plants respond to B deficiency with a cessation of growth at the growing tips, or meristems, in both the root and the shoot [13]. Meristems are groups of stem cells that eventually give rise to all postembryonic above and below the ground organs [14]. In Arabidopsis, one of the first phenotypic defects of B deficiency is the inhibition of root elongation through effects on the root apical meristem [15]. In maize, B deficiency also affects root growth and reduces crop yields [16,17]. Conversely, treatment of sunflower with BA stimulates adventitious root growth [18]. While there are benefits to increased B, too much is toxic and levels must be carefully controlled by the plant [19,20].

B is absorbed by roots as undissociated BA either passively via diffusion when B concentrations are adequate or high, or actively under B deficiency conditions by B import facilitators and export proteins [20]. *At*BOR1, the first characterized B transporter in Arabidopsis [21], exports borate out of the cell. *At*NIP5;1, belongs to the major intrinsic protein family and facilitates the import of BA [22]. B itself regulates uptake by controlling mRNA degradation and protein stability of the transporters [20]. The B responsivity of *At*NIP5;1 was recently used to develop a B sensor that responds to cytosolic B levels [23]. *BOR1* and *NIP5* homologs have been identified in several species including maize. The *AtBOR1* co-ortholog in maize is called *Zmrottenear* (*Zmrte*) and the *AtNIP5;1* co-ortholog in maize is called *Zmtassel-less1* (*Zmtls1*) [24,25,26].

One approach to decipher the role of B in plants is to induce B deficiency experimentally. Successful strategies to induce B deficiency have been to use inherently B deficient mutants, like the *Atnip5;1*, *Atbor1*, *Zmtls1*, or *Zmrte* mutants [21,22,24,25,26]. In recent years, chemical approaches to inducing B deficiency, such as the use of phenylboronic acid (PBA) have been published in petunia [27], apple [28], and Arabidopsis [29,30]. PBA lacks the third hydroxyl functional group in its chemical structure and therefore fails to generate bridging cis-diol complexes in the cell walls resulting in a B deficiency response [27]. The use of this chemical to induce B deficiency in maize has not been explored.

One reason the cellular functions of B have not been fully resolved is because B is present at low levels in plants [31]. Methods to visualize or quantify B, therefore, have limitations in sensitivity, standardization, reproducibility, and tissue destruction [2]. Past successes in quantifying B in soil and plant systems have mainly been through photometric methods quantified via UV-VIS [2,31]. Other quantification methods such as inductively coupled plasma-mass spectrometry (ICP-MS) or optical emission spectrometry (ICP-OES) have also been used for B quantification in soil and plant tissues, but are destructive and indicate only total B in digested tissue rather than spatial localization of B. Imaging and quantification by chemical methods such as fluorescence and low-energy x-ray fluorescence have not been successful as the natural concentration of B in plants is beneath their detection limits [32]. The same holds true for advanced microscopy techniques like scanning electron microscopy-electron energy loss spectroscopy or transmission electron microscopy (Matthes, McSteen, White, and Schaufflinger, unpublished results). Imaging B in plant systems has been accomplished by thermal neutron radiography in maize [33] and clover [34], but high resolution was not achieved. The use of a radiotracer could overcome the limitations of tissue destruction and resolution; however, there are significant challenges for the synthesis of a B radiotracer that works in plant systems. First, radionuclides of B are not suitable for incorporation into radiotracers as the longest half-life (t_½_) belongs to ^8^B at 770 ms [35], which limits synthesis, purification, and administration to plant systems. Second, since B is found as BA, a logical alternative would be to label oxygen (O) rather than B, but the longest lived radioisotope of O, ^15^O, has a t_½_ = 2 m [35]. Labeling hydrogen is not an alternative as it will be lost when B incorporates into the cell wall or reacts with other species in planta in the form of the borate anion.

**Figure 1:**
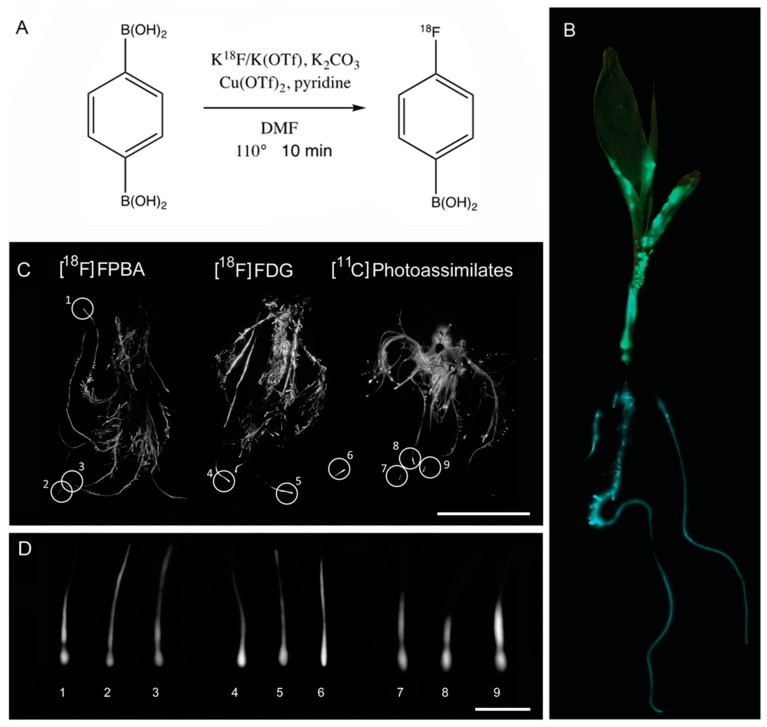
Synthesis and administration of a [^18^F]FPBA radiotracer to maize seedlings (inbred B73). (**A**) Single vial synthesis of [^18^F]FPBA from precursor 1,4-diboronic acid using a copper catalyst. (**B**) Radiographic image overlaid on visual image of five-days-old maize seedling after passive hydroponic administration of [^18^F]FPBA radiotracer. (**C**) Three-weeks-old maize root radiographic images under passive administration of [^18^F]FPBA, [^18^F]FDG, or [^11^C]CO_2_. Regions of interest appearing in circles are shown under greater magnification in panel D. (**D**) Primary root tips and elongation zones as they appear under radiographic imaging using [^18^F]PBA, [^18^F]FDG, or [^11^C]CO_2_. Scale bar in C = 15 cm and in D = 0.5 cm.

Here we test the ability to utilize fluorine-18 labelled 4-fluorophenylboronic acid ([^18^F]FPBA) as a radioactive tracer for B visualization and the consequences of the induction of B deficiency by PBA and unlabeled 4-fluorophenylboronic acid (FPBA) on maize root development. FPBA is a structural analog of PBA where a fluorine group is added across the aromatic ring from the BA group (Figure 1A) and is more amenable for radiolabeling as fluorine-18 [^18^F] chemistry is well established and relatively accessible.

## 2. Results

### 2.1. Synthesis of an [^18^F]FPBA Radiotracer

We hypothesized that PBA could be radioactively labelled in order to use it as a B radiotracer. As previously published, the radioactive [^18^F] fluoride can be incorporated onto the boranate ester of the PBA via a copper-mediated radiofluorination [36]. The t_½_ of [^18^F] is sufficiently long at 110 min enabling complex radiochemistry and use of the final radiotracer for imaging purposes [35]. In our experiments [^18^F]FPBA was synthesized in approximately 10 min from 1,4-phenyldiboronic acid precursor, with purification and quantification complete after an average of 90 min (Figure 1A). Specific activity, or measurement comparing radioactive labeled substrate to un-labeled substrate, of this radiotracer was 1.04 × 10^6^ Ci mol^−1^ as calculated to end of bombardment. This is a valid specific activity for tracing and imaging of B, as it is well beneath the expected concentration of B in plant tissues (10–100 mg kg^−1^ dry weight [2]). In contrast to published syntheses of [^18^F]FPBA [36], we added an additional purification step to the tracer production, although with limited success. A Chelex^®^ column removed much of the copper catalyst from the solution prior to separation on the high performance liquid chromatography (HPLC) semi-prep column.

Longer reaction times were explored up to 30 min but no increase in yield was observed after 10 min. Because of the nature of radioactivity, a synthesis should be as short as possible for maximization of product formed and specific activity of the final product. While [^18^F]-fluoride was produced on site via a cyclotron for the radiochemistry, it is possible to order the radionuclide and have it delivered if no access to a cyclotron exists, providing the proper licensing and safety protocols are followed. In the United States there now exist several regional distribution centers that can provide this resource.

### 2.2. [^18^F]FPBA localizes to the Primary Root and Lateral Root Initation Sites in Maize and Translocates to the Shoot Where It Localizes to Leaf Edges

Approximately 200 µCi of the synthesized [^18^F]FPBA tracer was hydroponically administered to the root system of maize plants (inbred B73) either at five days post-germination (Figure 1B and Figure S1) or three weeks post-germination (Figure 1C,D and Figure S2) by suspending the plant roots in the tracer-water. As a comparison, 2-deoxy-2-[^18^F]fluoro-d-glucose ([^18^F]FDG) and [^11^C]carbon dioxide ([^11^C]CO_2_, ^11^C t_½_= 20.4 min) were also administered to maize plants three weeks post germination (Figure 1C,D and Figure S2). [^18^F]FDG is used as a glucose mimic and [^11^C]CO_2_ can be used to image [^11^C]photoassimilates [37]. Autoradiographic images by phosphor film imaging were done as previously described [37]. Autoradiography is a static, two-dimensional radio-imaging technique. Our instrument parameters were set at 100 µm resolution. The images obtained can provide information about static B localization patterns in root and shoot tissues. In the radiographic images, the tissues with higher concentration of radiotracer uptake appear more intensely white while areas of no uptake appear black. It is apparent from the radiographic images that tracer activity was taken up and localized heterogeneously across the maize roots with greater uptake within the primary root and lateral root initiation sites along the primary root (Figure 1B–D and Figures S1 and S2). This differs greatly from the homogenous uptake exhibited by the [^18^F]FDG tracer, which was also passively taken up by the roots. The [^11^C]CO_2_ images resulting from passive tracer uptake through the leaf tissue show similar patterning of [^11^C]-photosynthates in the tips of the primary roots (Figure 1C,D and Figure S2). The [^18^F]FPBA radiotracer additionally translocated into the shoot, with signals being detected in the leaf margins (Figure 1B and Figure S1).

### 2.3. FPBA and PBA Elicit Similar Phenotypic Responses

#### 2.3.1. Germination of Maize kernels in PBA and FPBA

**Figure 2:**
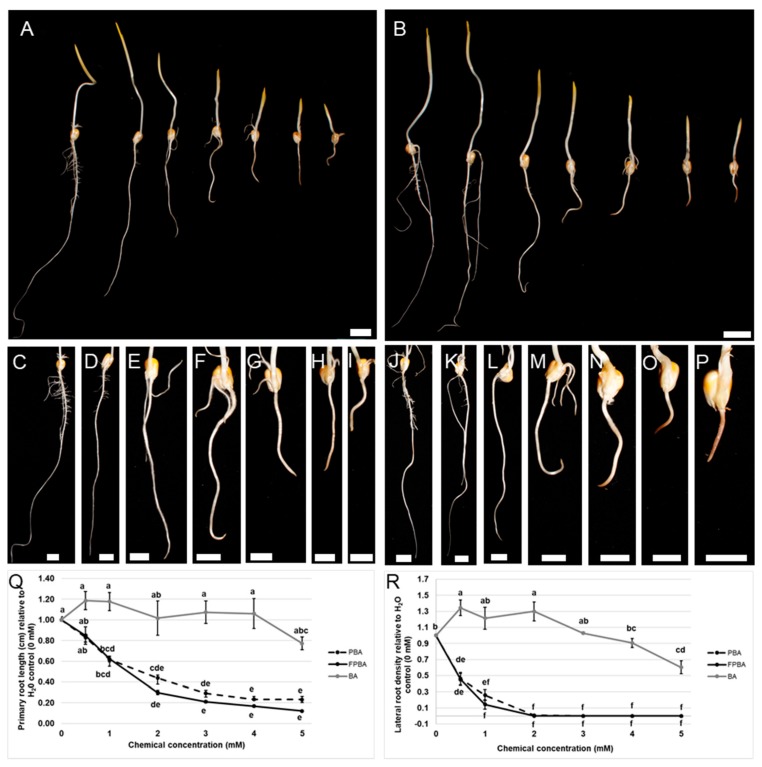
Phenotypes of maize root growth (B73 inbred) germinating in different concentrations of PBA and FPBA. (**A**) Maize B73 seedlings five days after germination in varying concentrations of PBA (from left to right: H_2_O, 0.5 mM, 1 mM, 2 mM, 3 mM, 4 mM, and 5 mM). (**B**) Maize B73 seedlings five days after germination in varying concentrations of FPBA (from left to right: H_2_O, 0.5 mM, 1 mM, 2 mM, 3 mM, 4 mM and 5 mM). (**C**–**I**) close up of B73 roots five days after germination in H_2_O (**C**), 0.5 mM PBA (**D**), 1 mM PBA (**E**), 2 mM PBA (**F**), 3 mM PBA (**G**), 4 mM PBA (**H**) and 5 mM PBA (**I**,**J**–**P**) Close up of B73 roots five days after germination in H_2_O (**J**), 0.5 mM FPBA (**K**), 1 mM FPBA (**L**), 2 mM FPBA (**M**), 3 mM FPBA (**N**), 4 mM FPBA (**O**), and 5 mM FPBA (**P**). (**Q**) Statistical analysis of primary root length of B73 seedlings grown in varying concentrations of PBA, FPBA or BA. (**R**) Statistical analysis of lateral root density of B73 seedlings grown in varying concentrations of PBA, FPBA, or BA. Different letters indicate statistical significance at *p* < 0.05 according to ANOVA and a post-hoc Tukey test. Scale bar in A, B = 2 cm and in C–P = 1 cm.

To enable the interpretation of the localization of [^18^F]FPBA in the radiotracer studies and since the effects of PBA on maize development have not been previously explored, we performed extensive testing of the effects of PBA and unlabeled FPBA on maize seedling development. We rolled maize kernels of the B73 inbred line into paper towels, incubated them in varying concentrations of PBA solution (0–5 mM) and allowed them to germinate for five days in the dark at 28 °C (Figure 2A,C–I). In comparison to kernels that were germinated in Millipore water, the PBA treated kernels developed into seedlings with shorter roots and coleoptiles (Figure 2C–I). We focused on the effects of PBA on root growth and found that the lowest concentration of PBA, which elicited a significant decrease in primary root length, was 1 mM (Figure 2Q). The effect of FPBA on maize root development was tested (0–5 mM FPBA), as done for PBA (Figure 2B,J–P). After five days in the dark at 28 °C a significant reduction was detected in primary root length in seedlings treated with FPBA compared to seedlings germinated in Millipore water, starting at 1 mM FPBA (Figure 2J–Q). The reduction in root growth with FPBA was not significantly different from PBA-treated B73 kernels, as determined by analysis of variance (ANOVA) followed by a Tukey test for multiple testing correction (Figure 2Q). Treatment with BA did not lead to a reduction in primary root length (Figure S3), rather a slight increase in primary root length was observed when seedlings were germinated in either 0.5 mM BA or 1 mM BA. Higher concentrations (5 mM) led to a reduction in primary root length, but not to the extent PBA did (Figure 2Q).

Next, the occurrence and density of lateral roots in B73 seedlings that were germinated in varying concentrations of PBA, FPBA, and BA were analyzed (Figure 2R and Figure S4). Concentrations of 2 mM or higher of both FPBA and PBA led to a total absence of lateral roots, while the tested concentrations of BA (0–5 mM) did not (Figure S4). The occurrence of a “nude” root phenotype appeared with lower concentrations of FPBA than PBA (compare 1 mM to 2 mM in Figure S4). We measured the area of the primary root where lateral roots occurred and counted all lateral roots that had developed to calculate the lateral root density per cm. Similar to the effects on primary root length, PBA and FPBA elicited similar effects on lateral root density (Figure 2R). Low concentrations (0.5 mM) of either chemical led to a significant decrease in lateral roots and germination of B73 seedlings in 2 mM PBA/FPBA or higher resulted in a lateral root density of zero (Figure 2R). In comparison, low concentrations of BA (0.5–2 mM) led to a slight, but significant increase in lateral root density (Figure 2R). The highest concentration of BA used in this study (5 mM), led to a significant decrease in lateral root density, though it did not reach zero, like the PBA and FPBA treatment (Figure 2R).

#### 2.3.2. FPBA Induces Rootless Arabidopsis Seedlings

To further validate that PBA and FPBA elicit similar effects, we treated developing Arabidopsis siliques with 50 mM PBA and 50 mM FPBA. PBA was previously shown to induce Arabidopsis seedlings with no primary root [29]. FPBA treatment of developing siliques led to the same rootless phenotype (Figure S5).

### 2.4. Mimicking of B Deficiency Symptoms by PBA/FPBA

#### 2.4.1. Co-Treatment of PBA/FPBA and BA Decreases the PBA/FPBA-Induced Inhibition of Primary Root Length in Maize

**Figure 3:**
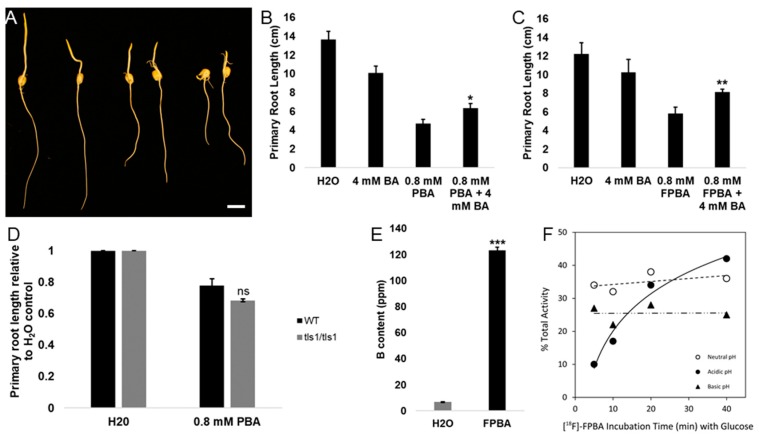
Mimicking of B deficiency symptoms by PBA/FPBA. (**A**) Rescue of PBA/FPBA induced primary root length defects by BA. From left to right: Seedlings germinated for five days in H_2_O, 4 mM BA, 0.8 mM PBA, 0.8 mM PBA + 4 mM BA, 0.8 mM FPBA, 0.8 mM FPBA + 4 mM BA. (**B**) Statistical analysis of primary root length of the rescue of PBA induced primary root length defects by BA as exemplified in (**A**). Shown is an individual experiment. (**C**) Statistical analysis of primary root length of the rescue of FPBA induced primary root length defects by BA as exemplified in (A); shown is an individual experiment. (**D**) Analysis of primary root lengths relative to H_2_O control for *Zmtls1* and normal seedlings grown for five days in either H_2_O or 0.8 mM PBA. (**E**) B measurements via ICP-MS in roots of seedlings germinated for five days in either H_2_O or 2 mM FPBA. (**F**) Binding affinity curves of [^18^F]FPBA to glucose at acidic, neutral, and basic pH conditions (pH = 4.0, 6.4, and 9.0 respectively). Scale bar in (A) = 2 cm. * *p* < 0.05, ** *p* < 0.01, and *** *p* < 0.001 (Student’s *t*-test). ns = non-significant.

In order to assess whether the observed reductions in primary root lengths were due to B deficiency, we tested whether the PBA/FPBA induced inhibition of primary root length can be alleviated by BA. Since PBA/FPBA are hypothesized to compete with BA for its binding sites, it was predicted that higher concentrations of BA in comparison to PBA/FPBA concentrations would be needed to rescue root growth. Therefore, B73 kernels in 0.8 mM PBA/FPBA were co-incubated with 4 mM BA (Figure 3A–C and Figure S6) and dark germinated for five days at 28 °C. The experiment was done four times with different seed batches. Treatment with 0.8 mM PBA or 0.8 mM FPBA led to significantly shorter primary roots in comparison to the H_2_O treatment (Figure S6). In the individual experiments co-treatments with BA led to significantly longer primary roots in comparison to PBA or FPBA treatment alone according to Student’s t- test (Figure 3A–C). A combined analysis of all individual experiments still led to slightly longer primary roots, yet no statistical significance was detected (Figure S6).

#### 2.4.2. The B Deficient Mutants *Zmtls1* and *Zmrte* Are Slightly More Sensitive to PBA Treatment Compared to their Normal Siblings

We next tested the sensitivity of the inherently B deficient mutants *Zmtls1* and *Zmrte* to PBA, by germinating mutant segregating lines in the B73 genetic background in 0.8 mM PBA (Figure 3D and Figure S7). Both mutants were previously shown to have reduced cytosolic B levels [24,25]. *Zmtls1* was also shown to have reduced RG-II crosslinking in the cell wall [24]. Germination in 0.8 mM PBA led to a significant decrease of primary root length in both normal and mutant siblings compared to the H_2_O control (Figure 3D and Figure S7). The same treatment led to a slightly stronger, yet not significant, reduction of primary root length in both *Zmtls1* and *Zmrte* mutants compared to their normal siblings (Figure 3D and Figure S7).

#### 2.4.3. FPBA Treatment Causes Enhanced Cellular B Levels in Roots

PBA has previously been shown to deliver B to the cell in Arabidopsis [38] and the induction of B deficiency symptoms is based upon PBA’s interference with cell wall crosslinking. In order to test whether FPBA behaves in the same way regarding B delivery to cells, we quantified B levels using ICP-MS in five-days-old roots of seedlings germinated from kernels that were treated with 2 mM F-PBA or H_2_O (Figure 3E). The amount of B measured was the summation of the concentration of both ^10^B and ^11^B isotopes naturally abundant. We found that B levels in FPBA-treated roots were about 18 times higher compared to the H_2_O control (Figure 3E). Other elements measured alongside B during the analysis (Figure S8) showed no significant differences in concentration between treatments with the exception of manganese, which showed significant reduction in the FPBA treated roots compared to H_2_O control roots.

#### 2.4.4. [^18^F]FPBA Binds cis-Diol Groups

To test the prediction that [^18^F]FPBA, like PBA and BA [27,39], has high affinity for covalent bonding with cis-diol groups, we performed an experiment to test the binding affinity with glucose which is a proposed binding site in vivo [39]. If a tracer is designed to mimic B behavior, it must bind cis-diols. Binding affinity was assessed with introduction of a cis-diol to the tracer at biologically relevant pH and separation of bound and unbound tracer was measured via radio-thin layer chromatography (radio-TLC). Glucose naturally exists in various physical conformations consisting of a linear structure, a six-membered ring pyranose structure, and a five-membered ring furanose structure, with the most common being pyranose in aqueous solutions. The furanose conformations of glucose also exist at low percentages and would have the strongest binding affinity for the [^18^F]FPBA based on the angles of the cis-diol bonds. The pH conditions can have an effect on the relative prevalence of the glucose conformers. Our data shows that as pH decreases, the total percent of bound radiotracer increases over time, while the neutral and basic pH show greater initial binding, with no enhancement of tracer binding over time (Figure 3F).

## 3. Discussion

B deficiency occurs in soils worldwide [4] and has negative effects on plant growth and performance. The underlying molecular mechanisms of how B deficiency limits plant growth are only marginally understood [5], in particular because of the limited availabilities of B imaging techniques. Here, we report the development of a B radiotracer in maize that is based upon the boronic acid FPBA. Boronic acids, like PBA, have been implicated in inducing B deficiency symptoms [27,29]. To our knowledge this is the first report of assessing the usability of a B deficiency mimic as a B radiotracer in plants and for assessing the effects of PBA on maize root development.

### 3.1. Development of a PBA-Based B Radiotracer in Plants

B belongs to a group of elements that does not possess any radioactive isotope with a t_½_ appropriate for isotopic labelling and autoradiography, since the longest t_½_ belongs to ^8^B at 770 ms [35]. A similar restriction is seen for O (longest t_½_ = 2 min). Because of these challenges in the possibility of labelling BA (the plant accessible form of B), we explored ways of labelling the B deficiency mimic PBA instead.

The most suitable radioisotopes for imaging an organic-based small molecule, like PBA, are those found before atomic number 10 (neon) on the periodic table [40]. Radioisotopes before carbon often have a t_½_ which is too short for synthesis, purification, and detection, while moving beyond into larger elements begins changing the chemical and physical properties of the small molecule. Such changes challenge the usefulness as a tracer of genuine chemical-of-interest behavior [41]. This makes ^18^F an attractive radioisotope for radiolabeling PBA.

Fluorine is more electronegative than the hydrogen it is replacing in PBA, but this is advantageous because the binding of BA and PBA in vivo is a result of deprotonation of the acidic hydrogens on the BA group for binding to cis-diols. Decreasing the pK_a_ of the BA group increases the binding affinity of the tracer for its substrate [42].

The radiotracer structure is not novel, as the replacement of a BA group with ^18^F on benzene rings has been an important aspect in medical research [43]. The radiotracer synthesis method described in this work is an automated version of a previously described manual synthesis [36]. Automated synthesis enhances safety for personnel and increases the level of radioactivity usable in the synthesis. Removal of the copper catalyst with a Chelex^®^ column and slight alterations to reagents and reaction times were also made.

The unique aspect of the radiotracer primarily lies in its application here for B imaging in live plant systems, which has not been previously explored. This radiotracer has advantages of a streamlined synthesis and hydroponic passive administration to plant. This allows avoidance of plant-stress responses that might otherwise have an impact on the observations and data. The stability and binding studies show [^18^F]FPBA has appreciable binding to the cis-diols like glucose across various pH values (Figure 3F) which means it will be able to bind to substrates across the entire spectrum of expected pH values in living plant systems and remain stable.

### 3.2. [^18^F]FPBA Localizes to the Root Tips and Lateral Roots in Maize and Translocates to the Shoot Where It Localizes to Leaf Edges

Autoradiography allows imaging of the localization of BA binding sites under the assumption that the radiotracer is intact and validly behaves just as BA in plant systems. The binding affinity and stability studies corroborate tracer physical integrity in vivo and the phenotypic studies suggest PBA behavior of the FPBA molecule in maize. The images can be overlaid on visual photographs to make more definitive conclusions concerning localization of the radiotracer and proposed purpose of higher B levels in such locations (Figure 1B and Figure S1).

This [^18^F]FPBA tracer shows different imaging locations and distribution from typical [^18^F]FDG tracers especially along the elongation zone of the primary and lateral roots, which indicates binding specificity of the tracer (Figure 1B–D and Figures S1 and S2). In five-days-old maize seedlings (Figure 1B and Figure S1) the [^18^F]FPBA radiotracer localized primarily to lateral roots and to specific locations in the young leaves, while uptake in three-weeks-old maize plants was mostly restricted to the root system (Figure 1C,D and Figure S2). The root tip and elongation zone displayed the most intense signal of the tracer with a region in between the two devoid of any signal (Figure 1D Numbers 1–3). This particular pattern was also observed in the [^11^C]-photoassimilate tracer (Figure 1D Numbers 7–9) and suggests that the location of B binding sites and areas of high metabolism and carbon at least partially overlap. PBA was previously used for the design of a fluorescent marker and used to visualize B binding sites in maize and sunflower roots [44]. In this study by Gluesenkamp et al. in 1997, the PBA marker localized to the elongation zone and spared the meristematic zone at the root tip, which for the authors was in line with previous reports stating only expanding tissues have a high demand for B [39]. It is known that root meristem activity is inhibited under B deficiency [45], suggesting a need for B in meristematic areas. The differences in localization between our tracer and the reported fluorescent marker are therefore likely due to structural differences of the two molecules. In five-days-old seedlings the [^18^F]FPBA radiotracer was additionally detected in the shoot at the edges of the leaves (Figure 1B and Figure S1). This pattern is reminiscent of patterns of B accumulation, since excess B typically accumulates at the leaf edges as well [46], therefore indicating that [^18^F]FPBA can be used to visualize sites of B occurrence in plants.

### 3.3. Induction of B Deficiency Symptoms by FPBA/PBA

One of the fastest reported responses upon B deficiency is the cessation of root elongation in both the primary and the lateral roots [13]. When maize B73 kernels were germinated in PBA or FPBA a significant reduction of primary root length and a significant reduction of lateral root density was observed in comparison to kernels that were germinated in H_2_O (Figure 2). The non-significant difference between PBA and FPBA treated seedlings indicates that the effects of PBA and FPBA on maize root development are comparable. This conclusion is also supported by the Arabidopsis experiment, where treatment of developing siliques with either PBA or FPBA leads to rootless Arabidopsis seedlings (Figure S5). Browning of the root tip and swelling of the roots was also observed, particularly with higher concentrations of either chemical used (Figure 2C–P). These findings are in good agreement with reported B deficiency symptoms in plant roots [13], suggesting that PBA and FPBA can also be used in maize to induce B deficiency symptoms as reported for other species [27]. On the contrary, germination of B73 maize kernels in BA led to the opposite effects, namely slightly longer primary roots and an increase in lateral root density (Figure 2Q,R and Figure S3). It further did not lead to a loss of lateral roots in any of the tested concentrations (Figure S4).

In all experiments FPBA was found to be slightly more potent in inducing phenotypic defects compared to PBA. Primary root length in B73 seedlings was shorter with lower concentrations of FPBA compared to PBA (Figure 2Q), a “nude” root phenotype occurred with lower concentrations of FPBA (Figure 2R and Figure S4), and the treatment of Arabidopsis embryos with FPBA led to two distinct phenotypes, suggesting either an earlier or prolonged effect of FPBA or slightly different targets of FPBA compared to PBA (Figure S5). Although there are exceptions, it is generally assumed that boronic acids with lower pK_a_s bind cis-diols more strongly. It is reasonable to speculate that the observed differences between PBA and FPBA (pK_a_s of 8.8 and 8.6 respectively [47]) are related to differences in their binding strengths such that FPBA binds cis-diols more strongly compared to PBA.

High concentrations of BA are toxic to plants and can lead to similar phenotypes to those observed under B deficiency. For example, excessive B has been reported to inhibit root growth in Arabidopsis [48]. While germinating B73 maize kernels in BA we also observed a reduction of primary root length in the highest BA concentrations likely because of toxicity. We measured, via ICP-MS, an 18-fold increase in B content in FPBA-treated roots compared to H_2_O control roots (Figure 3E), indicating that FPBA is taken up by the roots. Other elements measured were not significantly different (Figure S8). An exception to this was manganese, which showed significant reduction in FPBA-treated roots compared to H_2_O control (Figure S8). The reason for this observation remains the subject of future analysis.

Because of the increase in B level, the observed reduction in primary root length and the browning of the tissue in PBA and FPBA treatments could be due to toxic cytosolic B contents. However, our data suggest that the reduction of primary root length caused by FPBA/PBA is due to B deficiency rather than toxicity, because: (1) Addition of BA (4 mM) partially restored the PBA-induced (0.8 mM) reduction of primary root length (Figure 3A–C and Figure S6), and (2) the maize mutants *tls1* and *rte* appeared to be slightly more sensitive to PBA treatment (Figure 3D and Figure S7). The fact that addition of BA did not lead to a more severe reduction of primary root lengths in B73 seedlings makes it unlikely that the initial defects observed with PBA treatment are due to B toxicity, since the addition of BA would cause an even higher toxicity effect. The partial rescue in contrast of a full rescue by co-incubation of PBA with BA can likely be explained by the higher binding strength of PBA in comparison to BA because of their pK_a_s [47], which will shift the odds for competitive binding in favor of PBA. One could hypothesize that once PBA binds a B- binding site within the cell wall, BA’s lower binding strength cannot equally compete with PBA and the binding site is “lost” for BA. This scenario also explains why only high amounts of BA can partially rescue B deficiency defects induced by PBA as some available binding sites would be bound by BA. At the same time, BA concentrations cannot be increased infinitely as further increase in BA concentrations would lead to toxicity symptoms. This hypothesis is supported by previous studies with PBA, where PBA-induced defects were only partially or not at all rescued with added BA [27,29].

The responsivity to PBA is variable between different biological replicates (seed batches). Although the duration of the treatments was constant between the experiments, actual root lengths were variable between the individual experiments. Because of this variability the partial rescue of the PBA and FPBA induced primary root length defects by BA could reach statistical significance only by direct comparison (Figure 3B,C), but not when corrected for multiple testing (Figure S6).

In a second line of experiments to assess the induction of B deficiency in maize seedlings by PBA, inherently B-deficient maize mutants *tls1* and *rte* both appeared slightly more sensitive to PBA treatment in comparison to their respective normal siblings (Figure 3D and Figure S7) and displayed a higher reduction of primary root lengths when incubated in lower concentrations of PBA compared to their normal siblings. *Zmtls1* is a mutant of a B importer gene, co-orthologous to the Arabidopsis NIP5;1 B importer [22], and *Zmrte* is co-orthologous to the Arabidopsis BOR1 B exporter [21]. *Zmtls1* completely lacks the *tls1* gene, but can be rescued by addition of BA because of passive B transport [24,49]. The slight hypersensitivity of *Zmtls1* and *Zmrte* to PBA suggests that PBA likely is transported passively. Direct transport of PBA by TLS1 or RTE cannot be excluded and will need to be assessed in the future by, for example, oocyte transport assays [50]. The reported role of PBA in interfering with cell wall crosslinking could be a cause of the enhanced sensitivity of *Zmtls1* to PBA, as *Zmtls1* was shown to have a reduced RG-II-B crosslinking, increased RG-II monomers, and reduced cellular B content [24], which tempt the following speculations: (1) Less PBA is needed to occupy the remaining B-binding sites; and (2) the reduced cellular B content in *Zmtls1* cannot successfully compete with PBA. Similar speculations can be drawn for *Zmrte*, although reduced RG-II-B crosslinking or altered RG-II monomers have not been shown in this mutant [25]. It is interesting to note that the responsivity of PBA as detected by a reduction in primary root lengths was different in *Zmtls1* compared to *Zmrte* lines (comparing Figure 3D and Figure S7). PBA (0.8 mM) led to an approximately 40% reduction of primary root lengths in normal siblings in *Zmrte* segregating lines (Figure S7), which is comparable to the observations with B73 (Figure S6). The same concentration of PBA led to an approximately 23% reduction in primary root length in *Zmtls1* segregating lines (Figure 3D). Both mutants originated in different backgrounds than B73, yet have been backcrossed into B73 several times [24,25]. It is likely that remnants of the original backgrounds, specifically for *Zmtls1* segregating lines cause the difference in phenotypic expression. Further research is needed to elucidate and confirm underlying causes for the displayed hypersensitivity and variation between genotypes, which is beyond the scope of this report.

In conclusion, we report the development a [^18^F]FPBA B radiotracer to image B binding sites in maize. In good agreement with previous reports, our tracer localizes to particularly the tip, the elongation zone, and lateral roots in the maize root, showing a demand for B in these regions. Follow up phenotypic analyses with PBA and unlabeled FPBA suggest that the observed root defects are caused by B deficiency symptoms, likely because of an interference in B-dependent cell wall crosslinking. Our phenotypic data therefore support the usability of [^18^F]FPBA to image B binding sites in planta.

## 4. Materials and Methods

### 4.1. [^18^F]FPBA Radiotracer Production

Fluorine-18 was obtained in Oxygen-18 enriched water from a 16.4 MeV biomedical cyclotron (GE PETrace) with a starting activity of approximately 300 mCi. All chemistry was done remotely in a radiation safety hood. Activity was collected on a Waters Sep-Pak™ Light Accell Plus QMA Carbonate Cartridge (Milford, MA, USA) and eluted from the cartridge with 0.55 mL of a 0.05 M potassium trifluoromethanesulfonate (Aldrich, St. Louis, MO, USA) and 1.3 mM potassium carbonate solution. The eluted ^18^F^−^ was dried repeatedly with acetonitrile (Fisher, Hampton, NH, USA) 0.5 mL at a time at 100 °C in a reaction vessel suspended in an oil bath under argon gas. Dimethylformamide (Acros Organics) was added separately to 6.63 mg of benzene 1,4-diboronic acid (Oakwood Chemicals, Fair Lawn, NJ, USA), 0.810 mL pyridine (Acros Organics, Fair Lawn, NJ, USA), and 72.3 mg of copper (II) trifluoromethanesulfonate (Alfa Aesar, Haverhill, MA, USA). Once dissolved, 0.5 mL of the pyridine mixture, 0.10 mL of the copper mixture, and 0.05 mL of the benzene mixture were mixed together and this precursor solution was injected into the reaction vessel containing the dried residue of the fluorine solution. This solution was reacted at 110 °C for 10 min and mixed occasionally to form the unpurified [^18^F]FPBA. This solution was allowed to cool, then transferred through a SPE cartridge body containing 1 g of Chelex^®^ 100 resin (100–200 mesh, sodium form, Bio-Rad Laboratories, Hercules, CA, USA) held in place by a glass wool packing. This step was performed in an attempt to remove excess copper from the solution. The [^18^F]FPBA was rinsed off the resin and glass wool with 1 mL of 50:50 methanol:0.5 M HCl and collected in a vial upon elution.

The [^18^F]FPBA product was purified on a semi-prep Phenomenex Luna 5 µm column (250 × 10mm, 100A. PN:DN-00G-4448-N0) with mobile phase 70:30 acetonitrile:water plus 0.1% trifluoroacetic acid at 4 mL/min, and the UV-detector set to a 254 nm wavelength. The retention time of the product is approximately 15 min. Upon detection by the UV and radiation detectors the product was collected in 30 mL of water. It was then applied to a Waters Oasis HLB light 30 mg Sep-Pak™ cartridge for solvent removal and concentration. This cartridge was eluted with 1 mL of ethanol and evaporated to dryness with gentle heat argon. The product [^18^F]FPBA was then reconstituted first in 1 mL of water and a sample was removed for quality control testing.

Quality control of the radiotracer involved analytical HPLC as well as radio-TLC analysis of the final product. Upon resuspension of the radiotracer in 1 mL of water, 10 µL was injected onto the HPLC and analyzed on a Luna 5u PFP(2) 100A analytical column (2.50 × 4.6 mm, PN: 00G-4448-E0) and detected by radioactivity detector and internal UV detector. The mobile phase was as per preperative, flowing at 1 mL min^−1^. The final product spectra was compared to a cold FPBA standard and a phenyldiboronic acid standard. Percent labeled radiotracer was measured with radio-TLC on 3 × 10 cm amino-backed silica plates developed in a chamber of 2:1 methylene chloride:methanol mobile phase. Once developed, the distribution of activity was measured by a Bioscan AR-2000 radio-TLC reader and WinScan software. The spots where identified by a comparison of their location to a cold plate run with cold precursor and product standards.

### 4.2. Radiotracer Administration to Plants

After resuspension of the dried radiotracer in 1 mL of water, it was further diluted to 10 mL of DI water for the five-day old maize or 20 mL for the three-week old maize and swirled to adequately mix. Approximately 200 µCi of tracer was then hydroponically administered to the root system of the maize plants either at five days or three weeks post-germination by suspending the plant roots in the tracer-water. Tracer uptake was enhanced by illumination of the plant with LED lights to drive transpiration and water uptake. The plant and beaker were placed within a Plexiglass™ and lead lined radiotracer stall, held under slight negative pressure for safety. The plants were incubated with radiotracer for approximately 90 min before removal and autoradiography.

Tracer administration for the [^18^F]FDG comparison trials (500 µCi) was accomplished in an identical manner as the [^18^F]FPBA, but the administration for the [^11^C]-photosynthate trials were different. The [^11^C]CO_2_ was made on the cyclotron and administered to the maize seedling (~20 mCi) where it was passively administered in a gaseous bolus to a load leaf. This load leaf was placed within an air-tight gasketed cuvette in which the [^11^C]CO_2_ flowed in and out. After administration, the plant was allowed to metabolize the radiotracer for approximately 90 min at which point the plant was removed for imaging as done in previous work [37].

### 4.3. Autoradiography

Once removed from the radiotracer stall, and from either the cuvette or the beaker of aqueous radiotracer, the plant was patted dry and laid atop a phosphor imaging film. A visual (digital image) was taken of the plant prior to exposure to the film for overlay of the images later in Adobe Photoshop software. Original images prior to overlay can be found in the supplemental information (Figure S1). The plate was exposed overnight (~15 h), and then read on a Typhoon 9000 imager the following morning, imaging of the roots separately from the leaf tissue. In the case of the five-days-old seedling, the visual and radiographic image were overlaid in order to determine the location of the tracer more readily.

Imaging of the [^18^F]FDG plants was done in an identical manner to the [^18^F]FPBA but the [^11^C]-photosynthate trials had much shorter phosphor film exposure times because of higher activity levels of the radionuclide.

### 4.4. Chemical Kinetics on Maize Root Development

For each chemical and concentration, 10–15 kernels of the maize inbred line B73 were rolled into a paper towel. Each paper towel roll was incubated in a different concentration of either PBA (Sigma: P20009-250g), FPBA (Sigma: 417556-25g), BA, or water. Kernels were allowed to germinate in the dark for five days at 28 °C. After five days the seedlings were imaged using a Canon EOS Rebel T-6 camera. Primary root length and lateral root density was scored using ImageJ [51]. For lateral root density, the distance on the primary root from the kernel until the last lateral root was measured and all lateral roots were counted. Afterwards the number of lateral roots was divided by the distance to yield lateral root density per cm. Experiments were repeated at least three times. Averages, standard deviations, and significance (Student’s T-Test) were calculated using Microsoft Excel. Analysis of Variance with a post-hoc Tukey Test to correct for multiple testing was done in R. For the connecting letter report the multcompView package in R was used [52].

For the PBA/FPBA sensitivity experiment on *Zmtls1* and *Zmrte* seedlings, 3:1 WT:mutant sibling lines of either mutant were used. Per treatment and experiment, 45 kernels were rolled up in paper towel rolls. Kernels in each paper towel roll were germinated in the dark for five days at 28 °C. Pictures of the seedlings were taken with a Canon EOS Rebel T-6 camera and shoot tissue for each seedling was dissected, DNA isolated and genotyped for *Zmtls1* and *Zmrte* as described in [24,25,26].

### 4.5. Boron Measurements in Maize Roots

Roots of seedlings grown in H_2_O or 2 mM FPBA were harvested at five days and the roots were collected and frozen for quantification of B via ICP-MS. The root samples were freeze-dried for 24 h in a Thermo Fisher Freezone 1 freeze dryer (Labconco Corp., Kansas City, MO, USA) and then gently ground with mortar and pestle to homogenize. An approximately 0.2 g representative sample was taken from each sample and microwave-digested (Milestone Ethos Plus, SK-12 rotor, Milestone Inc., Shelton, CT, USA) in 2.50 mL of 14 N HNO_3_ at 190 °C for 25 min. Once digested, the samples were diluted to 50 mL using 18 MΩ-cm water, internal standard was added, and then samples were analyzed via ICP-MS (NexION 300X, KED mode, PerkinElmer Inc., Branford, CT, USA) for endogenous B concentration.

### 4.6. Glucose Tracer Binding Across pH

Tracer affinity for glucose across different pH levels was tested using the tracer made as described in Section 4.1. Once eluted off the HLB Sep-Pak™, the 1 mL of ethanol tracer was dried under gentle heating and argon gas and re-suspended in 3 mL of DI water. Three separate 1-mL aliquots were taken and acidic, neutral, and basic 10 mM glucose solutions were added to the tracer water aliquots respectively. The neutral glucose solution was 3 mL total volume with no additions and was a pH of 6.4. The acidic glucose was made by taking another 3 mL of 10 mM glucose and adding 30 µL of 0.03 M HCl which was at a pH of 4. The basic glucose solution was made with a 3-mL aliquot of 10 mM glucose with 30 µL of 0.027 M NaOH, bringing the pH to 9. Once the three glucose solutions were made and mixed with the radiotracer, samples were taken at time points of 0, 10, and 20 min and percent bound tracer was investigated with radio-TLC. The three pH glucose-tracer solutions were spotted onto amino-backed silica plates at the origin and developed in 2:1 methylene chloride:methanol until complete when radio-TLC analysis was completed to determine percent bound tracer to glucose across pH and time.

### 4.7. Chemical Treatment of Arabidopsis Thaliana Siliques

Developing siliques of Arabidopsis were treated with either PBA, FPBA, or H_2_O as described in [29]. After ripening of the seeds, all seeds treated with a respective chemical were harvested, sowed on a H_2_O agar plate, stratified for two days at 4 °C, and let to germinate at 28 °C under continuous light. The presence of rootless seedlings was scored.

## Supplementary Materials

Supplementary materials can be found at https://www.mdpi.com/1422-0067/21/3/976/s1.

## Abbreviations

^o^ C	Degrees Celsius
t_1/2_	Half life
Arabidopsis	*Arabidopsis thaliana*
B0_3_^3-^	Borate anion
B	Boron
BA	Boric acid
C	Carbon
CO_2_	Carbon dioxide
[^x^Y]	Radioactive element Y
FPBA	4-Fluoro-phenylboronic acid
H	Hydrogen
HPLC	High performance liquid chromatography
ICP-MS	Inductively coupled plasma mass spectrometry
ICP-OES	Inductively coupled plasma optical emission spectrometry
O	Oxygen
PBA	Phenylboronic acid
TLC	Thin Layer chromatography
*tls1*	*tassel-less1*
*rte*	*rotten ear*
*Zm*	*Zea mays*
