# Supplementary material for: Assessment of a 18F-Phenylboronic Acid Radiotracer for Imaging Boron in Maize
Source: Int J Mol Sci. 2020 Feb 1;21(3):976. doi: 10.3390/ijms21030976 (PMC7037850; doi:10.3390/ijms21030976)
Supplement: Supplementary file 1 [file ijms-21-00976-s001.pdf]

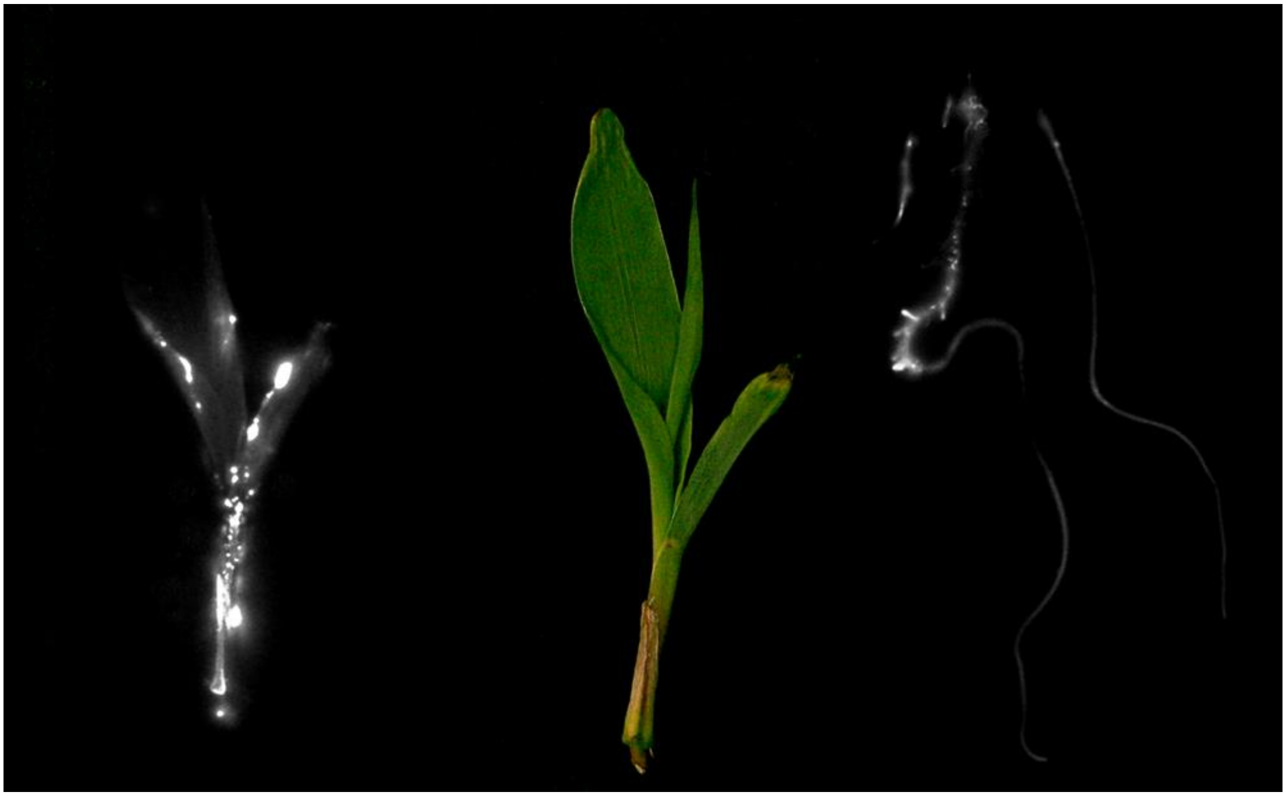

Figure S1 Original images for composite image in Fig. 1B.

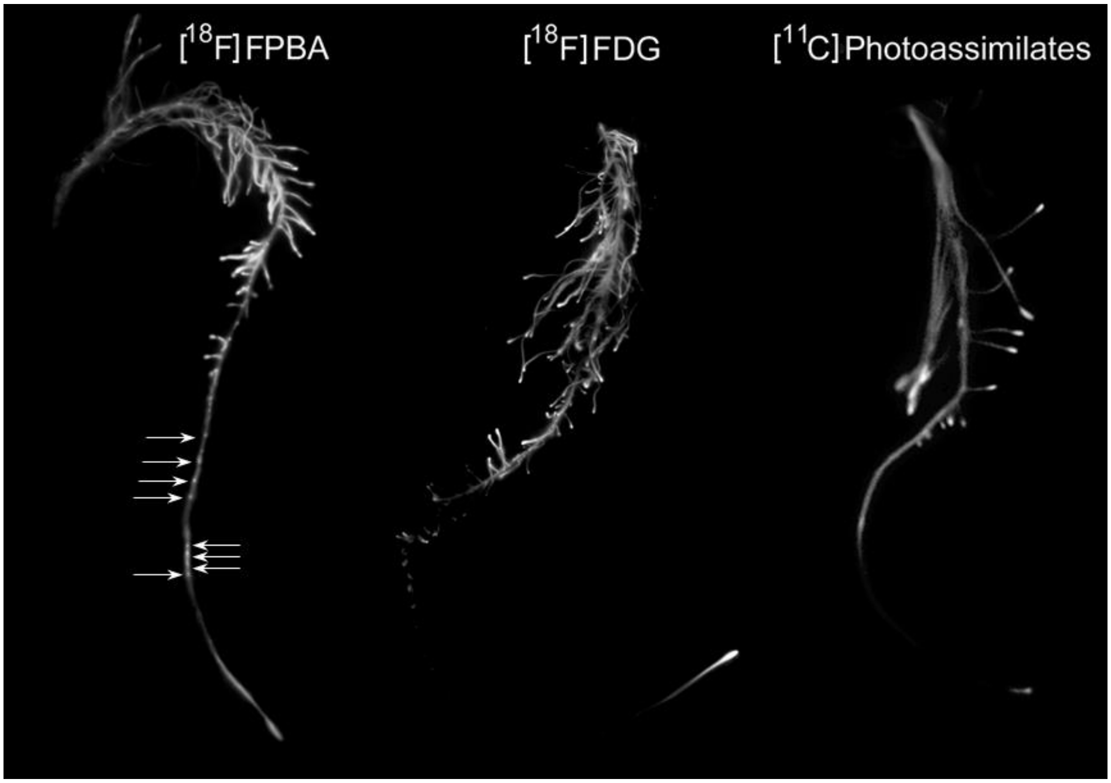

Figure S2 Isolated root images, showing localization of [^{18}F]FPBA, [^{18}F]FDG and [^{11}C]Photoassimilates in roots of three weeks old maize plants (inbred B73) as indicated.

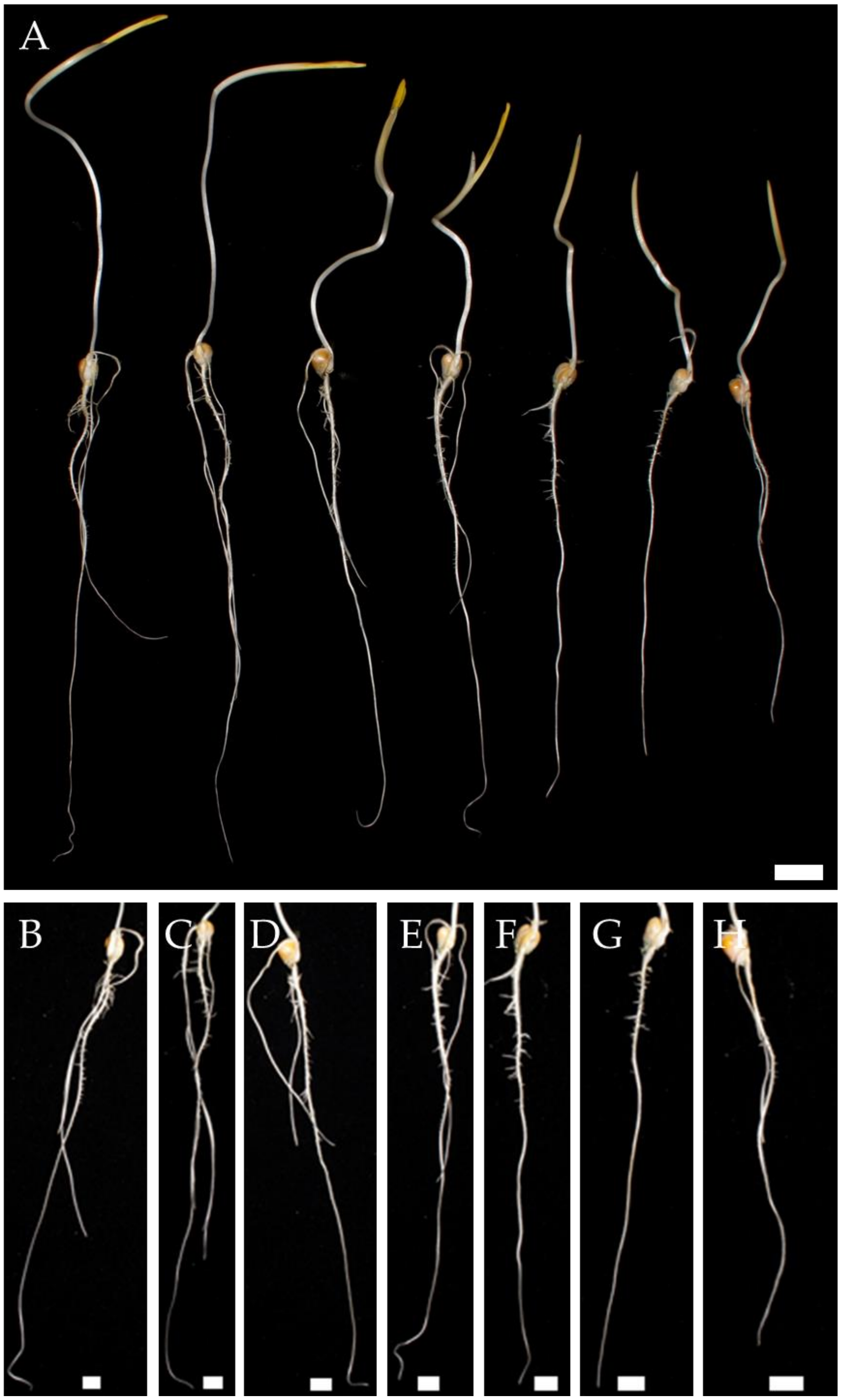

Figure S3 Phenotypes of maize B73 root growth germinating in different concentrations of BA. A) Maize B73 seedlings five days after germination in varying concentrations of BA (from left to right: H_2O, 0.5 mM, 1 mM, 2 mM, 3 mM, 4 mM and 5 mM). B) – H) close up of B73 roots 5 days after germination in H_2O (B), 0.5 mM BA (C), 1 mM BA (D), 2 mM BA (E), 3 mM BA (F), 4 mM BA (G), and 5 mM BA (H). Scale bar in A = 2 cm, and B-H = 1 cm.

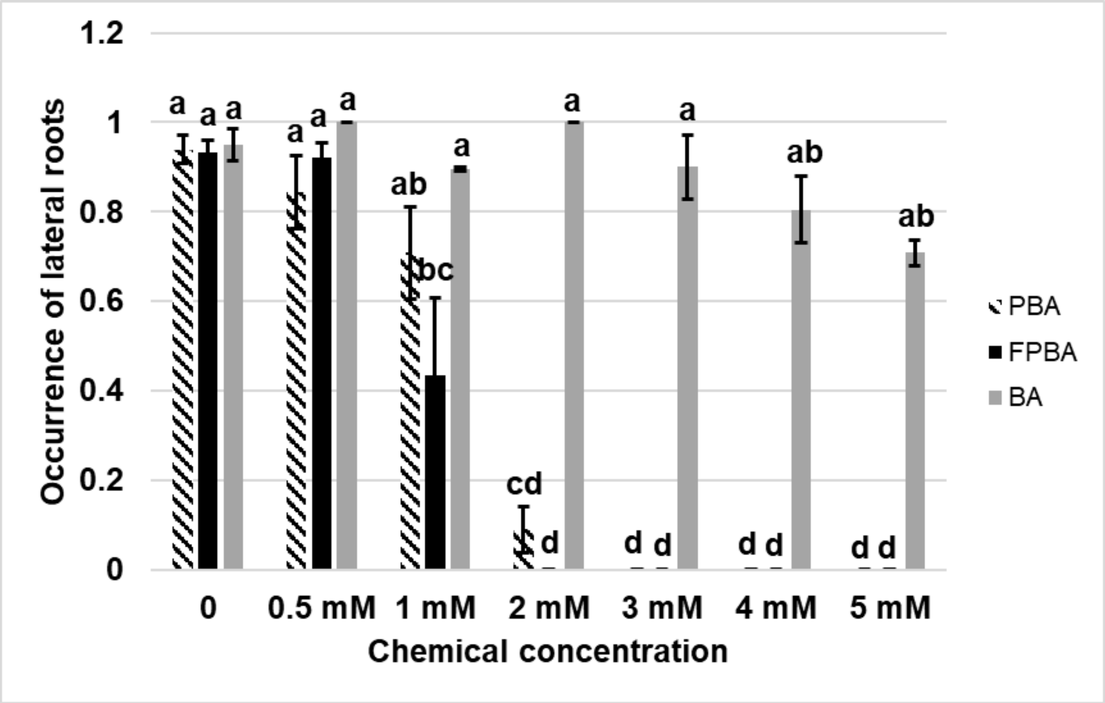

Figure S4 Percentages of B73 maize seedlings with lateral roots five days after germination in different concentrations of PBA, FPBA and BA. Different letters indicate statistical significance at $p < 0.05$ (ANOVA with posthoc Tukey test). Error bars indicate standard error.

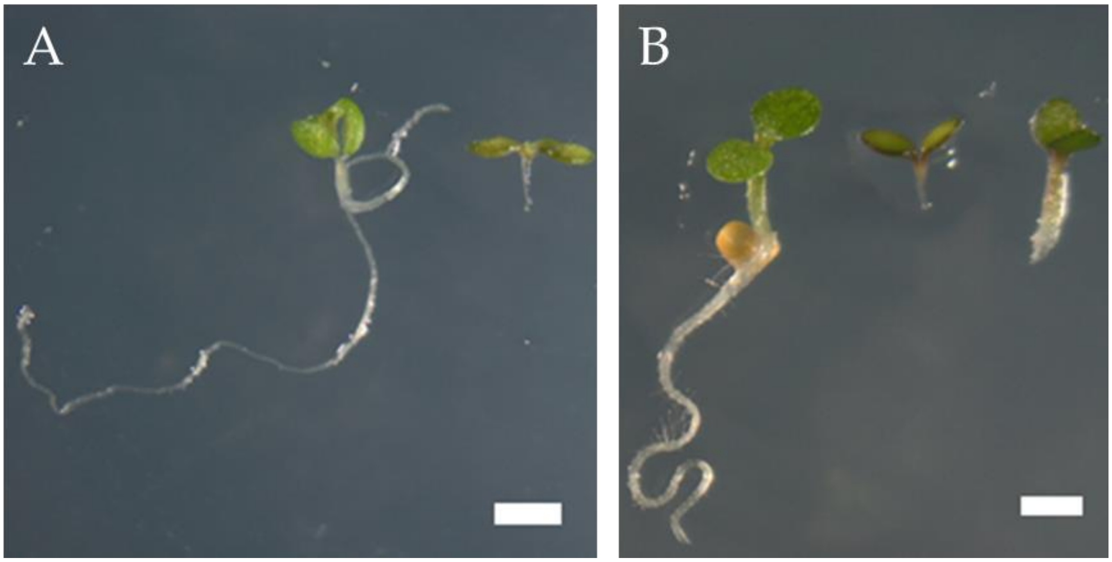

Figure S5 Phenotypes of seedlings resulting from PBA and FPBA treated Arabidopsis siliques. A) Phenotypes of Arabidopsis seedlings resulting from siliques treated with 50 mM PBA (left: normal, right: rootless phenocopy). B) Phenotypes of Arabidopsis seedlings resulting from siliques treated with 50 mM FPBA (left: normal, middle: rootless phenocopy, right: seedling with no root, but elongated hypocotyl). Scale bars = 1 cm.

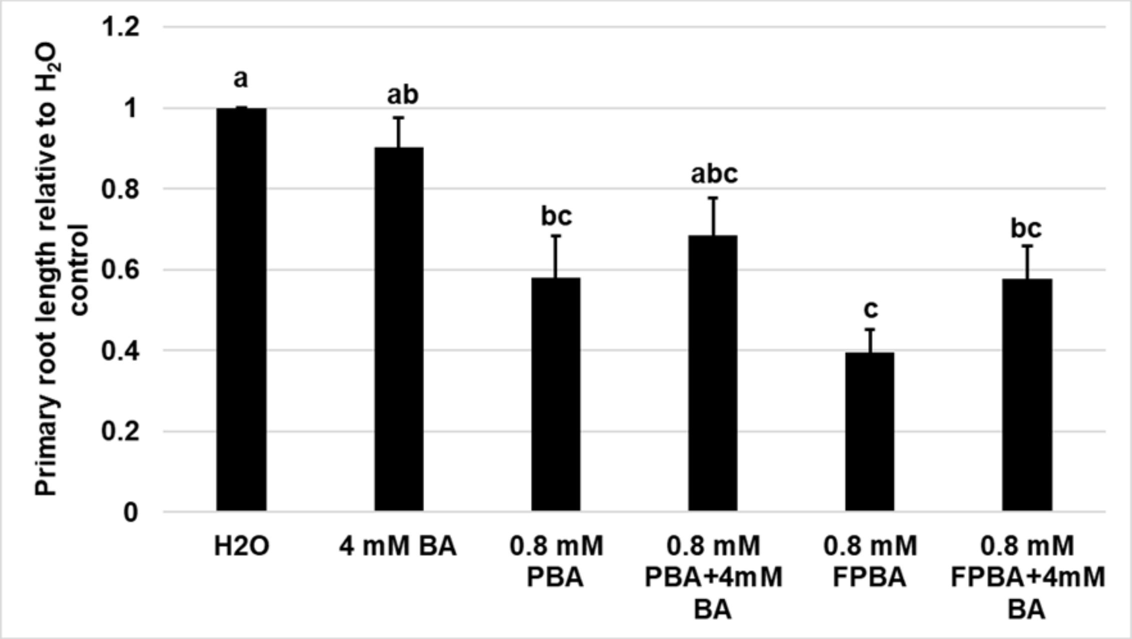

Figure S6 Analysis of primary root lengths of B73 maize seedlings germinated for five days in H_2O, 4 mM BA, 0.8 mM PBA, 0.8 mM PBA + 4 mM BA , 0.8 mM FPBA, and 0.8 mM FPBA + 4 mM BA (averages of four individual experiments). Different letters indicate statistical significance at $p < 0.05$ (ANOVA and posthoc Tukey). Error bars depict standard error.

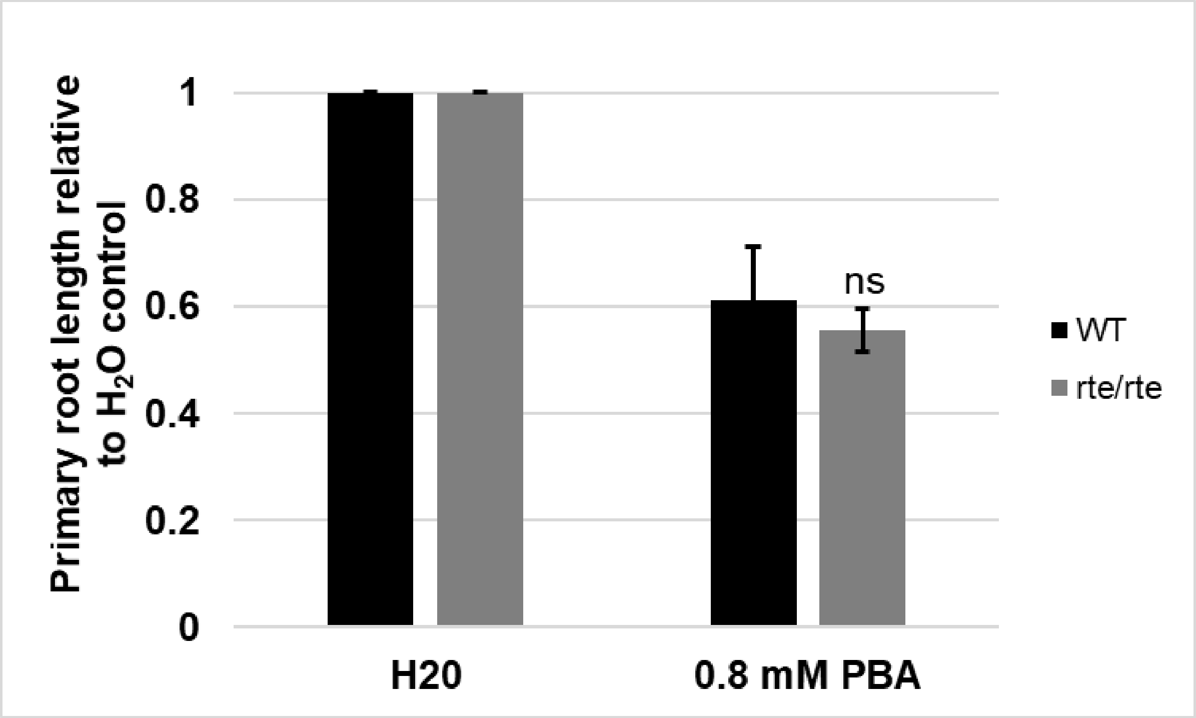

Figure S7 Primary root length relative to water control of WT and *rte/rte* maize seedlings that were germinated in 0.8 mM PBA in comparison to H_2O. Error bars indicate standard error.

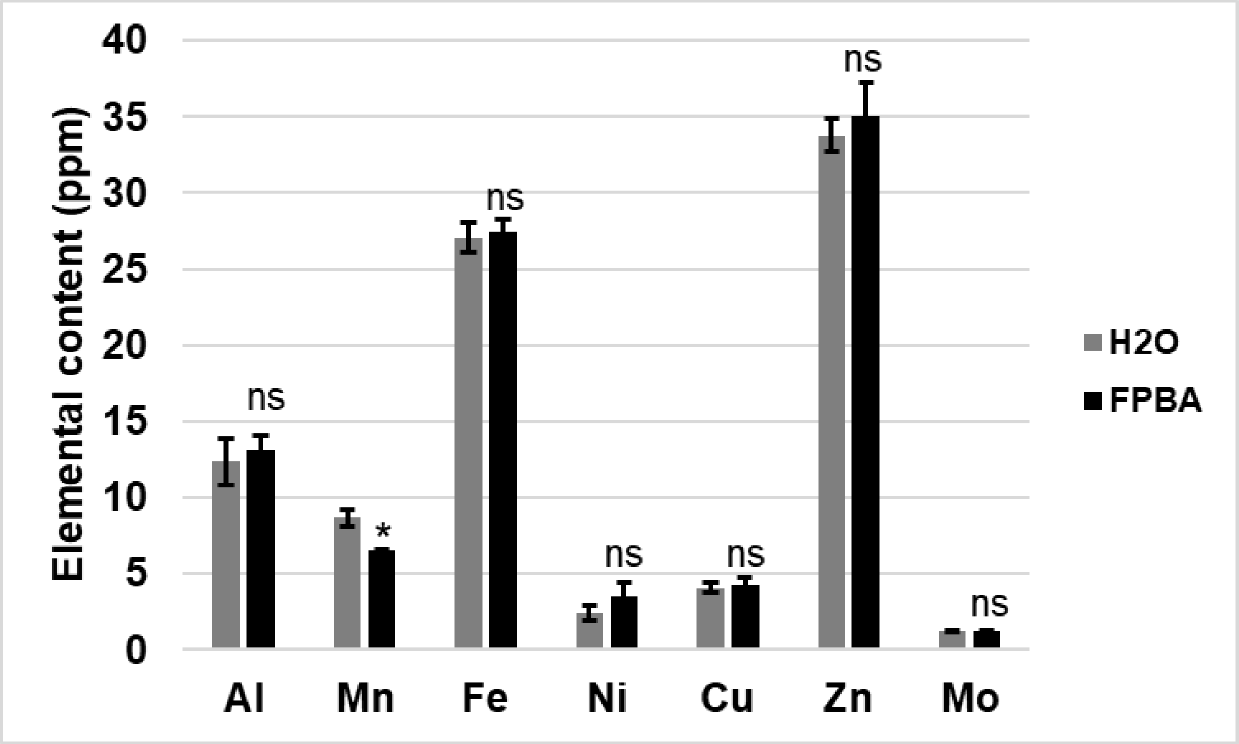

Figure S8 ICP-MS analysis of different elemental contents in roots of five days old maize seedlings (B73 inbred), that were germinated in 2 mM FPBA or H_2O. * indicates statistical significance at $p < 0.05$ (Student's t-test). Error bars indicate standard error.
